# The emerging role of lysine succinylation in ovarian aging

**DOI:** 10.1186/s12958-023-01088-4

**Published:** 2023-04-20

**Authors:** Meiling Le, Jia Li, Dalei Zhang, Yuan Yuan, Chong Zhou, Jinxia He, Jian Huang, Liaoliao Hu, Tao Luo, Liping Zheng

**Affiliations:** 1 School of Public Health and Basic Medical College, Nanchang University, Nanchang, 330006 Jiangxi China; 2 Key Laboratory of Reproductive Physiology and Pathology of Jiangxi Province, Nanchang University, Nanchang, 330006 Jiangxi China; 3 Basic Medical College and Institute of Life Science, Nanchang University, Nanchang, Jiangxi 330031 China; 4 Jiangxi Provincial Key Laboratory of Preventive Medicine, Nanchang University, Nanchang, Jiangxi 330006 China

## Abstract

**Background:**

Ovarian aging is a process of decline in its reserve leading to ovary dysfunction and even reduced health quality in offspring. However, aging-related molecular pathways in the ovary remain obscure. Lysine succinylation (Ksuc), a newly post-translational modification (PTM), has been found to be broadly conserved in both eukaryotic and prokaryotic cells, and associated with multiple pathophysiological processes. There are no relevant reports revealing a link between the molecular mechanisms of ovarian aging and Ksuc.

**Methods:**

The level of Ksuc in ovaries of aged and premature ovarian insufficiency (POI) mice were detected by immunoblotting and immunohistochemical. To further explore the role of Ksuc in ovarian aging, using in vitro mouse ovary tissue culture and an in vivo mouse model with changed Ksuc level.

**Results:**

Increased Ksuc in ovaries of aged and POI mice and distribution of Ksuc in various types of mice ovarian cells and the high level of Ksuc in granulosa cells (GCs) were revealed. Histological assessments and hormone levels analyses showed that the high Ksuc level down-regulated the ovarian index and the anti-Müllerian hormone (AMH) and estrogen levels, and increased follicular atresia. Moreover, in the high Ksuc groups, the terminal deoxynucleotidyl transferase-mediated dUTP-biotin nick end labeling (TUNEL) intensities and the expression of Cleaved-caspase-3 increased and the expression of B-cell lymphoma-2 (Bcl-2) decreased together with positively-expressed P21, an aging-related marker. These results suggest that ovarian aging is likely associated with alteration in Ksuc.

**Conclusion:**

The present study has identified Ksuc in mouse ovary and found that high Ksuc level most likely contributes to ovarian aging which is expected further investigation to provide new information for delaying physiological ovarian aging and treating pathological ovarian aging.

**Supplementary Information:**

The online version contains supplementary material available at 10.1186/s12958-023-01088-4.

## Introduction

With the postponement of marriage and childbirth, female reproductive aging and the related problems are becoming increasingly serious. The ovaries affect women’s physical health at all stages of life. Ovarian aging involves the decline in the quantity and quality of follicles, and even increases the risk of diseases including cardiovascular disease, diabetes and cancer [1, 2]. Therefore, exploring the mechanism of ovarian aging to “rejuvenate” the ovary may help with multiple conditions. Ovarian aging refers to the negative correlation between ovarian function and age, and premature ovarian insufficiency (POI) or premature ovarian aging, which is pathological ovarian aging, a disorder of ovarian function in females below the age of 40 years [3]. Existing studies suggest that ovarian aging is influenced by multiple factors, such as age, genetics and environment [1]. However, the molecular mechanisms underlying ovarian aging are not well understood. With the development of molecular techniques, the molecular mechanisms of ovarian aging continue to be studied in depth, including mitochondrial dysfunction, epigenetic alterations and metabolic abnormalities [2]. Among them, the role of post-translational modification (PTM) in the progression of ovarian aging has been well studied [4–6].

Lysine succinylation (Ksuc) is a conserved, novel PTM commonly found in eukaryotes and prokaryotes, which can affect protease activity and gene expression. It occurs by the transfer of succinyl groups from succinyl-COA to amino acid residues of the protein to be modified by succinyltransferases, with lysine being the most easily-modified amino acid due to its high number and unstable residue. The charge of the lysine residue is changed from + 1 to -1 by the addition of the succinyl group, resulting in large structural and functional changes of the protein undergoing succinylation, which may have a more profound impact on the protein than lysine methylation (Kme) and lysine acetylation (Kac) [7–9]. Proteomic studies on Ksuc have shown that succinylated lysines are highly enriched in both mitochondrial (Mt) and extra-Mt proteins involved in cellular physiological functions, including the tricarboxylic acid (TCA) cycle, mitochondrial respiration and fatty acid synthesis. Thus, the process is associated with the development of multiple diseases like cardiovascular disease and cancer [9–11]. Furthermore, our previous study revealed that the global Ksuc in mouse ovary increases significantly with advancing age [12]. Thus, in this study we further evaluated the effect of excessive Ksuc on ovarian function to reveal the relationship between Ksuc and ovarian aging.

In this study, we characterized global Ksuc in ovaries of mice with POI and aging mice via immunoblotting and immunohistochemistry with a qualified pan-anti-succinyl-lysine antibody. The co-factor of Ksuc, succinyl-CoA, was identified in mouse ovary. Moreover, we investigated the influence of excessive Ksuc on ovarian aging in mice administered with disodium succinate through its effects on steroid hormone levels and follicular development. The results of our study provide the first report of Ksuc as a likely important regulator of ovarian aging in mice, enriching the network of ovarian aging mechanisms and potentially contributing to the promotion of reproductive health and enhanced fertility in females.

## Methods

### Animals

Animal care and use were conducted in accordance with the guidelines of Experimental Animal Science and Technology Center of Nanchang University (Permit Number: SYXK (gan)-2021–0004, Table S1). Female mice (KM) aged 21 days and 3, 12 and 17 months (3 M, 12 M, 17 M) were housed in specific pathogen-free conditions at the Nanchang University Experimental Animal Center.

Mouse ovary culture in vitro: The ovaries were stripped from 21-day-old female KM mice and cultured as previously described [13]. A 6-well Millicell Standing Inserts (4.0 μm; Millipore Corporate, Billerica, MA; PICM03050) floating on 2 ml Waymouth MB 752/1 culture medium (Sigma, St. Louis, MO) previously equilibrated to 37 °C. Five ovaries were placed on each insert. After 24 h incubation in complete medium, the ovaries were treated with 40 mM NaCl (S8210, Solarbio, Beijing, China, control group, *n* = 5 mice) or 40 mM succinate (HY-W015410, MedChemexpress, New Jersey, USA, succinate group, *n* = 5 mice) for 48 h. NaCl and succinate were dissolved in double distilled H_2_O at an initial concentration and added to culture medium to achieve the desired final concentration. The quality of the ovaries culture was assessed by the pale red, smooth and shiny ovaries under microscope, and pale yellow and clear medium after 48 h incubation with NaCl or succinate.

Mouse ovary administered in vivo: The 3 M KM mice were randomly assigned into the above groups and injected with the corresponding drugs into the ovaries following 2 weeks of feeding. After the 21^st^ day of the administration, the weights of the bodies and ovaries of the mice were measured. Mouse eyeball serum collection: The collected eyeball blood was centrifuged at 12,000 g for 15 min, and the supernatant was collected. The mouse ovaries were photographed with a measuring ruler to compare the size of the ovaries in the two groups.

Induction of mouse POI model: The 3 M KM mice were intraperitoneally administered with 70 mg/kg cytoxan (CTX) (C0768, Sigma-Aldrich, St. Louis, MO, USA) and 12 mg/kg busulfan (BU) (B2635, Sigma-Aldrich) once a week for two weeks according to previous studies [14, 15]. Validation of successful model construction was shown in Fig. S1.

### Histological analysis of ovarian tissue and ovarian follicle count

Ovaries were collected and fixed with 4% paraformaldehyde to prepare as paraffin blocks. Sections of 5 μm were obtained and stained with a hematoxylin–eosin (HE) staining kit (AR1180-100, Boster Bioengineering Co., Ltd., Wuhan, China). Digital images were acquired using a microscope (FV3000, Olympus, Tokyo, Japan) and analyzed using ImageJ software (Version 1.52, National Institutes of Health, Bethesda, MD, USA). Follicular stages were determined according to the criterion described previously [16].

### Immunohistochemistry (IHC)

IHC was carried out on 5 μm sections of paraffin-embedded tissue. After slides baking, dewaxing, rehydration, the antigen retrieval was performed. The primary antibodies used for IHC were anti-cleaved caspase 3 (#9664, Cell Signaling Technology, Massachusetts, USA) and anti-Ksuc (PTM401, PTMBio, Hangzhou, China) diluted 1:100 in 5% bovine serum albumin (BSA) (SW3015, Solarbio). The secondary antibody and visualized stain for peroxidase was performed using the DAB Substrate Kit (PV-6000D, ZSGBbio, Beijing, China), and slides were counterstained with hematoxylin (AR1180-100, Boster). To confirm the validity and reliability of reagents used in IHC staining especially the specific antibody, the negative control groups were set (Fig. S2A).

### Immunofluorescence (IF)

After slides baking, dewaxing, rehydration, the antigen retrieval was performed. The ovarian sections were incubated with 0.1% Triton X-100 for 1 h at room temperature. After blocking with 5% BSA (SW3015, Solarbio) for 1 h, the samples were incubated with the primary antibodies overnight at 4 °C. The primary antibody was anti-pan-Ksuc (1:100, PTM401, PTMBio). The secondary antibody (fluorescein isothiocyanate conjugated goat anti-rabbit IgG, 1:100, E-AB-1014, Elabscience, Wuhan, China) was incubated with the samples for 1 h in the dark at room temperature. The cell nuclei were stained with DAPI Staining Solution (C1005, Beyotime, Shanghai, China). To confirm the validity and reliability of reagents, negative control was set (Fig. S2B). Fluorescence images were obtained using a fluorescence microscope (Olympus, Tokyo, Japan).

### Terminal deoxynucleotidyl transferase-mediated dUTP-biotin nick end labeling (TUNEL) assay

Ovarian sections were labeled with TUNEL reagents according to the product brochures (MK1014-100, Boster). The sections were counterstained with hematoxylin to visualize the nucleus. To confirm the validity and reliability of reagents, negative control and positive control were set (Fig. S3). The percentage of TUNEL-positive cells (%) in the mouse ovary was analyzed using ImageJ software.

### Western blotting

Total proteins were extracted from mouse ovaries using radio-immunoprecipitation assay (RIPA) buffer (R0010, Solarbio) with phenylmethylsulfonyl fluoride (PMSF) (ST506, Beyotime, Shanghai, China) and protease inhibitor cocktail (HY-K0010, MedChemexpress). Equal amounts (20 μg) of proteins were separated by 12% sodium dodecyl sulfate polyacrylamide gel electrophoresis (SDS-PAGE) and then electrophoretically transferred to polyvinylidene fluoride (PVDF) membranes (IPVH00010, Sigma-Aldrich). Subsequently, the membranes were hatched overnight at 4 °C with primary antibodies (Table S2). At room temperature, the horseradish peroxidase (HRP)-conjugated secondary antibody (1:10,000) was incubated on the membrane for 1 h. Anti-mouse IgG HRP-conjugated goat (SA00001-1) and anti-rabbit IgG HRP-conjugated goat (SA00001-2) secondary antibodies were purchased from Proteintech (Rosemont, IL, USA). Analysis of relative levels of target protein was conducted by quantifying the gray value of the target bands (as for the quantification of Ksuc, representative bands with differences were selected to quantify) normalized to those detected by β-actin using ImageJ software.

### RNA extraction and quantitative real-time PCR (qRT-PCR)

Total RNA was isolated using RNAiso Plus (9108, Takara Bio, Kyoto, Japan). The cDNA was acquired with the PrimeScript RT kit (6210A, Takara Bio, Kyoto, Japan) according to the manufacturer’s instructions. Then, the synthesized cDNA was used as the template for qRT-PCR. Primers are shown in Table S3. The qRT-PCR was performed in triplicate using SYBR Premix Ex TaqTM Kit (MF013, Mei5bio, Beijing, China). Relative expression values were calculated with the 2^−ΔΔCt^ method. Values of gene expression were calculated as the means of three replicates and β-actin was used as a reference.

### Hormone assay

Serum estradiol (E2) (E-EL-0150c, Elabscience) and anti-Müllerian hormone (AMH) (E-EL-M3015, Elabscience) levels in the serum were measured using an enzyme-linked immunosorbent assay (ELISA) kit according to the manufacturer’s instructions.

### Statistical methods

All data are represented as mean ± standard error of the mean (SEM) and the data from the experiments were analyzed by one-way analysis of variance (ANOVA) for multiple groups and Student's *t*-test for two groups with GraphPad Prism 8.0.2. (California, USA).

## Results

### The global Ksuc in mouse ovary increases significantly with advancing age and is distributed in various types of mouse ovarian cells

**Fig. 1:**
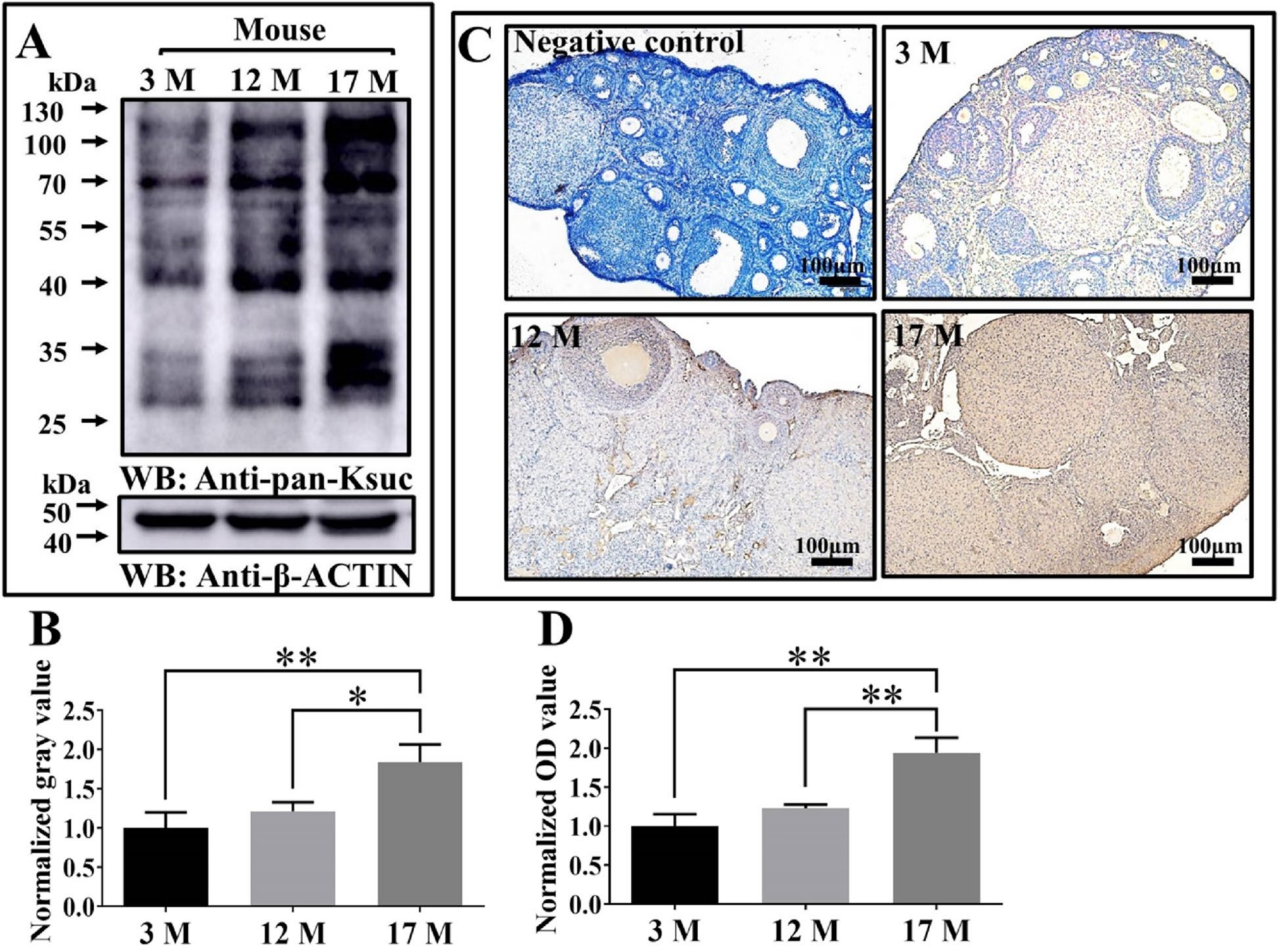
The level of lysine succinylation (Ksuc) in ovaries of mouse with different ages. **A** and **B** The changing mouse ovarian pattern of global Ksuc with increasing age, mice aged 3, 12 and 17 months (3 M, 12 M, 17 M). **C** and **D** The representative immunohistochemical (IHC) staining and semi-quantification of Ksuc in mouse ovaries with different ages (*n* = 3). Scale bar = 100 μm. Statistical significance was determined by one-way ANOVA. * *P* < 0.05, ** *P* < 0.01

To investigate whether Ksuc is correlated with ovarian aging, the level of Ksuc was detected by immunoblotting and immunohistochemical (IHC). The global Ksuc in the mouse ovary increased extensively with increasing age for physiological ovarian aging (Fig. 1A and B). The results of IHC with the anti-pan-Ksuc antibody showed a significant increase from 3 months old to 17 months old (Fig. 1C and D). Additionally, we observed that Ksuc occurs in various types of ovarian cells according to the results of IHC (Fig. 1C) and IF (Fig. S4A).

### Increased Ksuc in ovaries of POI mice

**Fig. 2:**
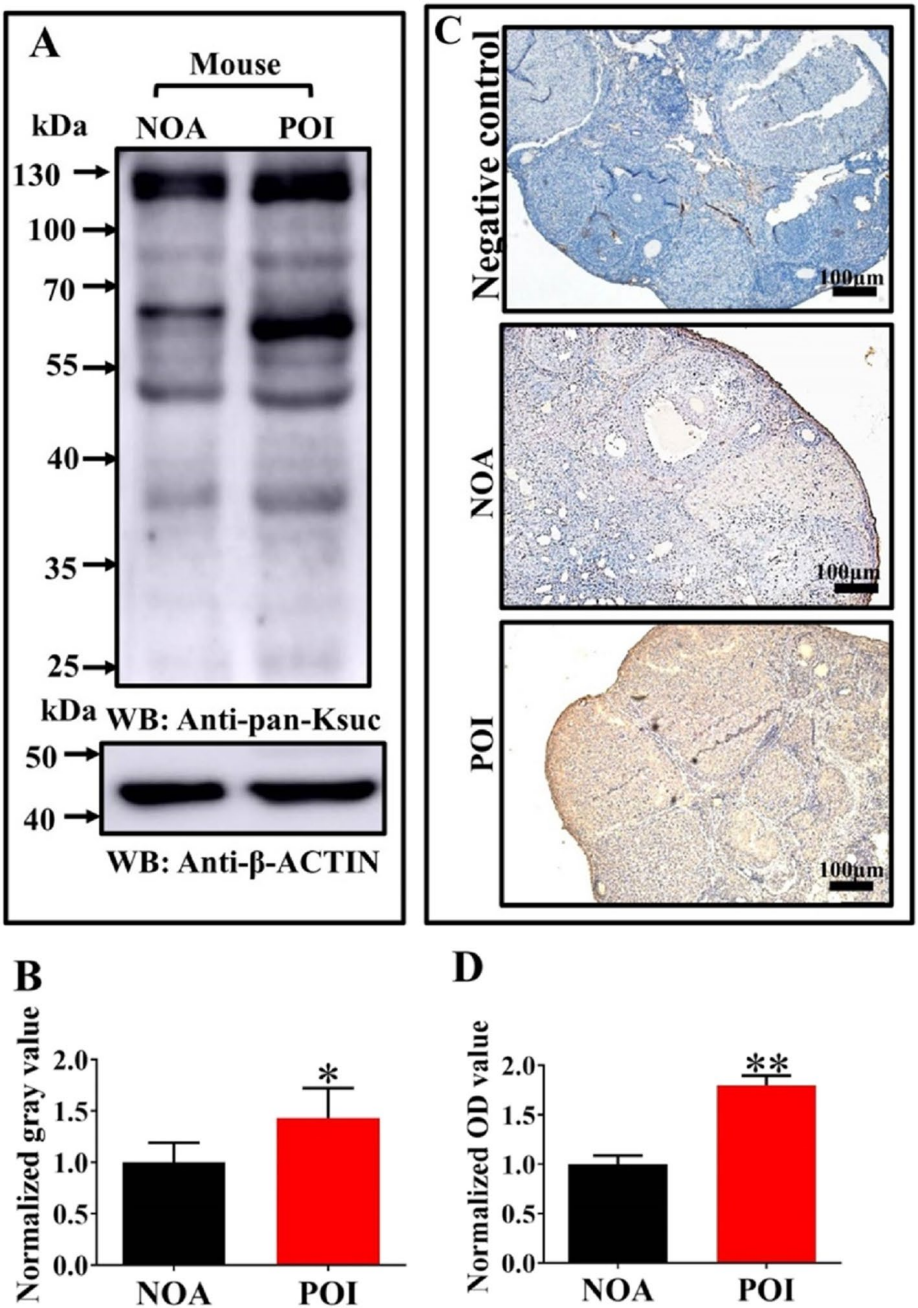
The levels of Ksuc in ovaries of premature ovarian insufficiency (POI) mice. **A** and **B**, Comparison of global Ksuc in ovaries between POI and normal ovarian aging (NOA) mice. **C** and **D**, The representative IHC staining and semi-quantification of Ksuc in POI and NOA mice (*n* = 3). Scale bar = 100 μm. Statistical significance was determined by Student's *t*-test. * *P* < 0.05, ** *P* < 0.01

Increased Ksuc in the ovary of mouse POI model caused by CTX and BU was detected (Fig. 2A and B). The results of IHC also revealed that the level of Ksuc in the ovary of POI mice was also higher than that of normal ovarian aging (NOA) mice (Fig. 2C and D). Thus, Ksuc may have a strong correlation with the development of ovarian aging.

### Global increase of mouse ovarian Ksuc promoted cell apoptosis in ovaries cultured in vitro

**Fig. 3:**
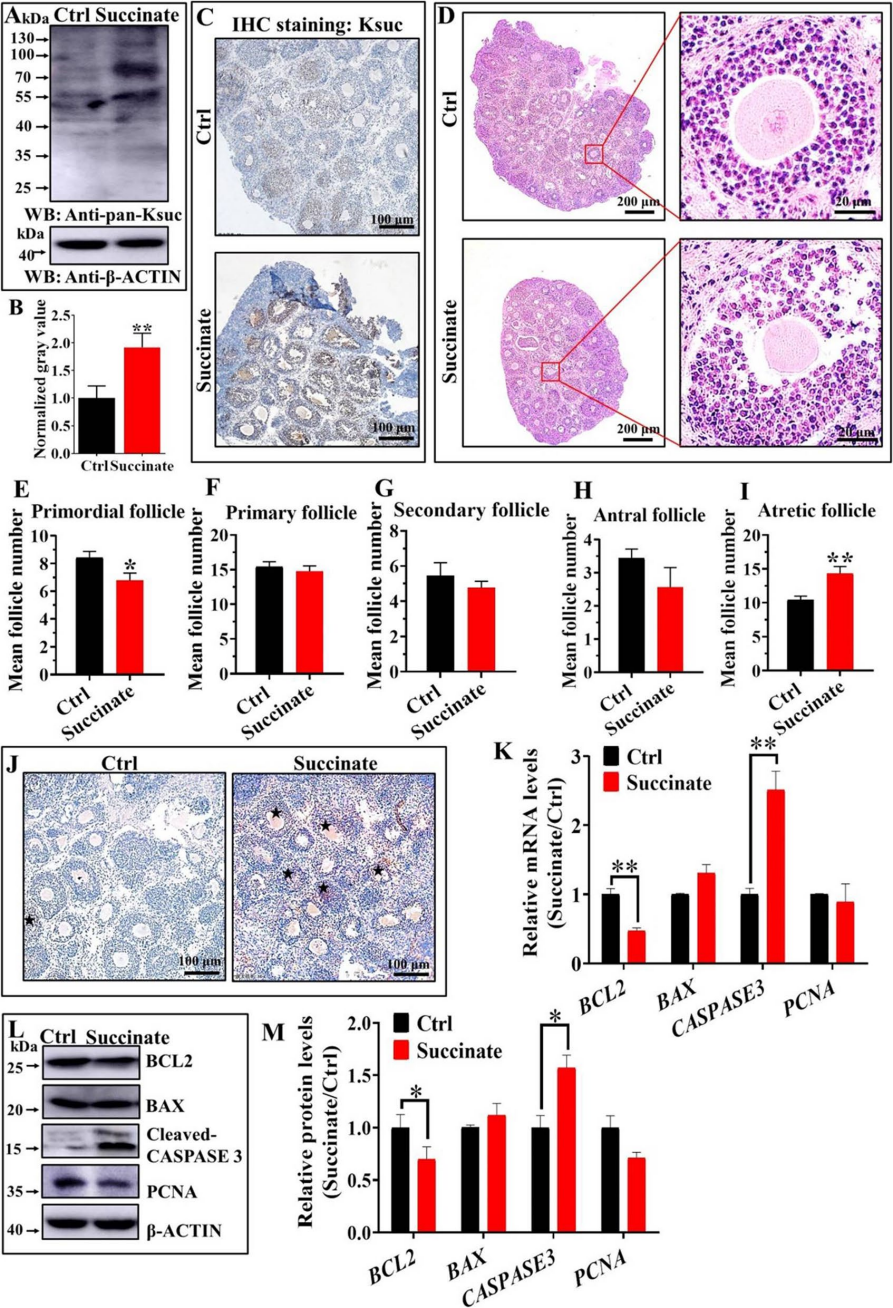
Effects of succinate on mouse ovary cultured in vitro. **A**-**C** Succinate elevated the global Ksuc level of mouse ovary cultured in vitro. Bar = 100 µm. **D** The hematoxylin–eosin (HE) staining was performed to evaluate the effects of high level of Ksuc on the ovarian histomorphology of mouse. Bar = 200 µm (left), 20 µm (right). **E**–**I** Summary of follicle represented as the mean of three ovarian sections per mouse from the ovaries of two groups. **J** Transferase-mediated dUTP-biotin nick end labeling (TUNEL) assay showed an increased staining in ovarian cells in the succinate group as compared with the control group (Bar = 100 µm). Asterisks indicate TUNEL-positive cells. **K**-**M** Both quantitative real-time PCR (qRT-PCR) and western blot verified that B-cell lymphoma-2 (Bcl-2) significantly decreased and Cleaved-caspase 3 significantly increased in succinate-treated ovaries, whereas BCL2-associated X (Bax) and proliferating cell nuclear antigen (PCNA) did not change significantly in both protein and mRNA levels. * *P* < 0.05, ** *P* < 0.01

Given the systemic increase of Ksuc in aged and POI ovaries, we further investigated the effect of elevated Ksuc level on mouse ovarian function. It has been reported that disodium succinate can elevate the level of Ksuc by increasing succinyl-CoA (a cofactor of Ksuc) [17]. Our results revealed that in vitro addition of succinate could elevate the Ksuc level in the mouse ovary, which confirmed that succinyl-CoA also works efficiently in the mouse ovary (Fig. 3A and B). Meanwhile, the elevation was specific to Ksuc, since succinate had negligible influence on structurally similar PTMs such as Kac (Fig. S4B) and lysine malonylation (Kmal) (Fig. S4C). Interestingly, succinate could elevate the Ksuc level in ovarian cells especially GCs (Fig. 3C). To further analyze the effect of excessive Ksuc on the morphological state of the ovaries, HE staining was conducted. As shown in Fig. 3D, the GCs in the ovaries of the succinate administration group were more sparsely arranged around the follicles compared to the control group. Follicle counting was performed after HE staining. In mouse ovary of succinate group, the number of primordial follicle obviously reduced and the number of atretic follicles increased significantly, but there were no significant differences of the number of primary follicles, secondary follicle and antral follicle between two groups (Fig. 3E-I). However, 40 mM of NaCl had no obvious effect on the ovaries (data not shown), which excluded the possibility that the effects of succinate were caused by osmotic changes in the bath solution.

In order to explore the effect of Ksuc on ovarian cell apoptosis and proliferation abilities, we performed TUNEL staining in ovaries cultured in vitro and determined the expression of B-cell lymphoma-2 (Bcl-2), BCL2-associated X (Bax), cleaved-caspase 3 and proliferating cell nuclear antigen (PCNA). As shown in Fig. 3J, we observed TUNEL positivity at all time-points in preovulatory follicles and mainly in the GCs. Furthermore, a clear decrease in Bcl-2 mRNA and protein levels was detected in the succinate treatment group when compared to the control group (Fig. 3K–M). The analysis also revealed that the expression of Cleaved-caspase 3, which serves as the terminal effector molecule in many types of apoptosis [18], increased markedly in the succinate group compared with the control group (Fig. 3K–M). However, the Bax and PCNA mRNA and protein levels in the ovary cultured with succinate were not significantly different from those in the control group (Fig. 3K–M). These results suggest that the mechanisms causing increased apoptosis in response to high Ksuc level in mouse ovarian GCs might be mediated by the Bcl-2 and Caspase-3.

### High Ksuc level could affect the physiological state of mouse ovary in vivo

**Fig. 4:**
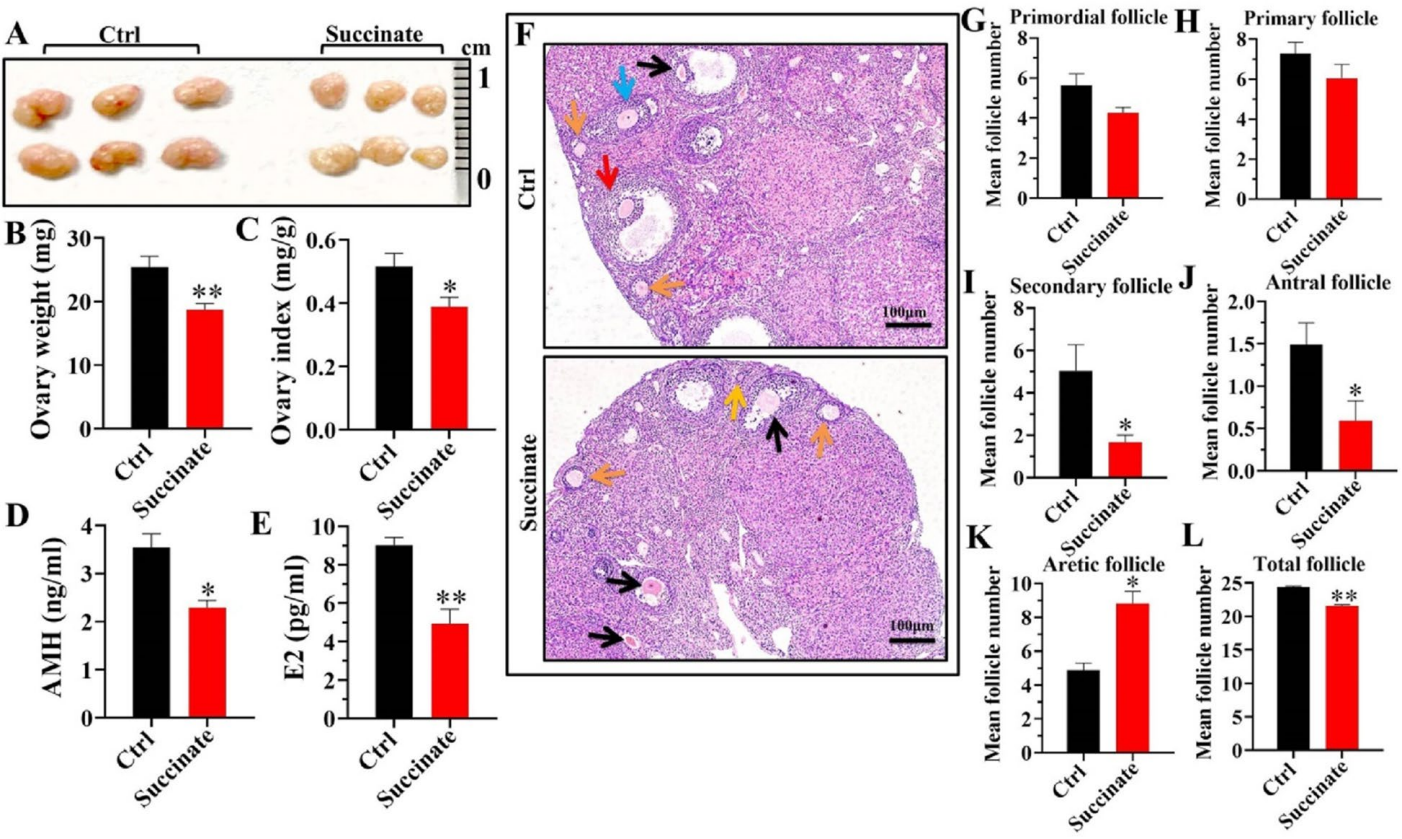
Effects of in situ ovarian administration of succinate on mouse ovarian function. **A** Ovarian appearance after in situ ovarian injection with NaCl (Ctrl, 40 mM) and succinate (40 mM). **B** and **C** Ovary weight (mg) and ovary index (ovary weight / body weight, mg/g). **D** and **E** Effects of high Ksuc level on serum anti-Müllerian hormone (AMH) and estradiol (E2) concentrations in mice after ovarian administration in vivo. **F** Histopathological examination and follicle counts of ovaries. The following different types of ovarian follicles were observed: primordial follicle (yellow arrow), primary follicle (green arrow), secondary follicle (blue arrow), antral follicle (red arrow), atretic follicle (black arrow). Scale bar: 100 μm. **G**–**L** Summary of follicle represented as the mean of three ovarian sections per mouse from the ovaries of two groups. * *P* < 0.05, ** *P* < 0.01

Based on the above data, we further confirmed the effect of excessive Ksuc on the mouse ovary in vivo. As shown in Fig. 4A and B, the ovarian size and weight of mice (18.74 ± 0.95 mg) markedly decreased in the succinate administration group compared with the control group (25.44 ± 1.66 mg). Meanwhile, the ovary index (ovary weight / body weight) in the succinate administration group was also lower than that in the control group (Fig. 4C). Interestingly, we found that the serum levels of AMH and E2 in the succinate group were significantly decreased (Fig. 4D and E), along with increased atretic follicles and reduced secondary, antral and total follicles (Fig. 4F–L). These results suggest that high Ksuc level likely suppresses the ovarian reproductive and endocrine functions.

### Excessive Ksuc could promote mouse ovarian aging by regulating follicular development and ovarian cell apoptosis and proliferation in vivo

**Fig. 5:**
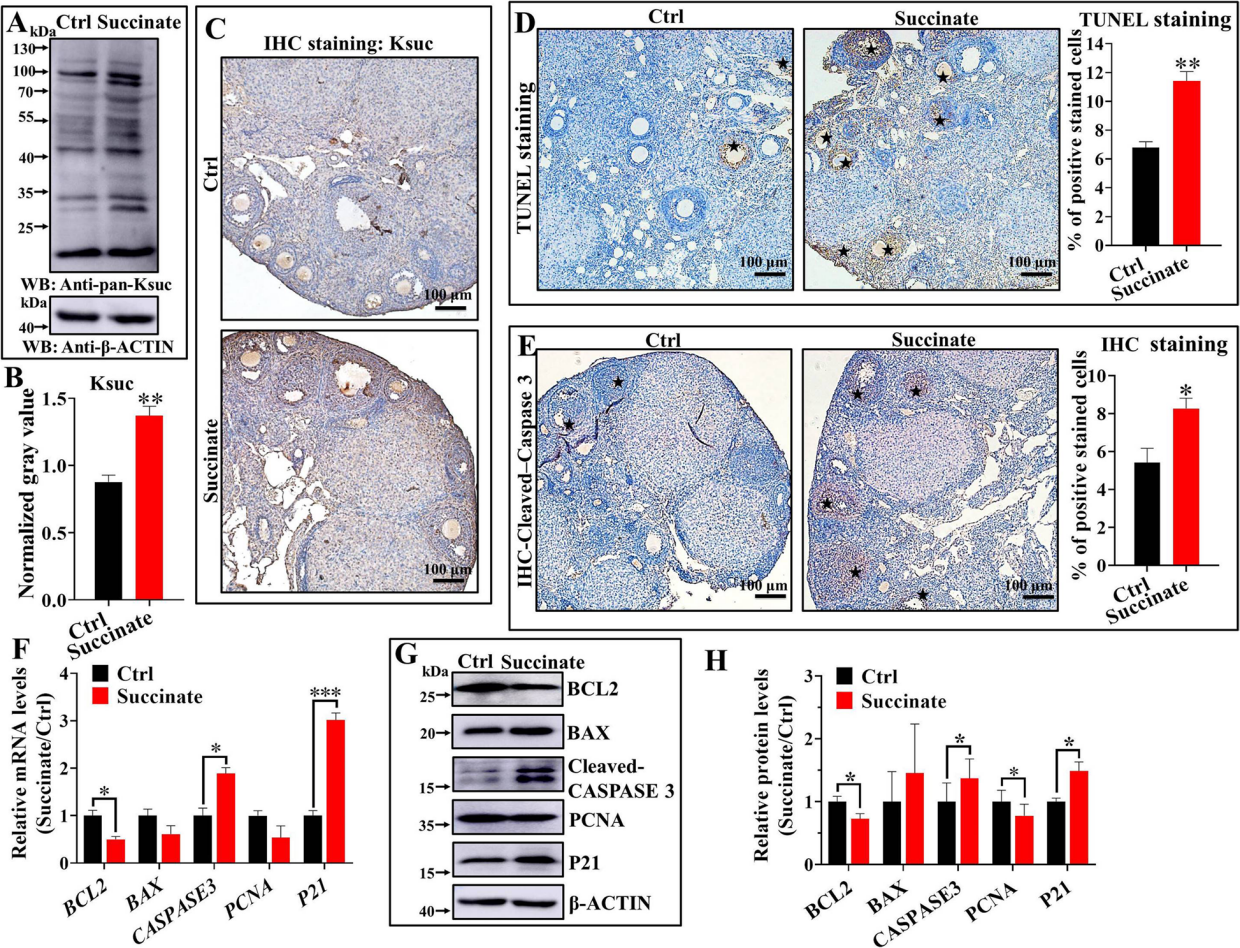
Ovarian cell apoptosis and impaired gene expression in succinate-administrated mice. **A**-**C** Western blot analysis of ovarian global Ksuc level in succinate-administrated mice in vivo. Scale bar: 100 μm. **D** and **E** TUNEL and IHC data on Cleaved-caspase 3 assay show an increase of apoptosis in ovarian cells mainly GCs of succinate-administered ovaries as compared with the control group. Asterisks indicate TUNEL-positive cells. Scale bar: 100 μm. The qRT-PCR (**F**) and western blot (**G**) and (**H**) analyses for mouse ovarian cell apoptosis, proliferation and P21, an ovarian aging-related marker. Error bars: SEM; (*n* = 5 mice / group). * *P* < 0.05, ** *P* < 0.01, *** *P* < 0.001, according to two-tailed Student’s *t*-test

Importantly, the injection with succinate in situ in the ovaries was able to elevate the global Ksuc level of mouse ovarian cells in vivo as in vitro (Fig. 5A-C). Based on this, we would argue that the above phenotypes and the following molecular level changes associated with ovarian dysfunction are due to the high level of Ksuc.

Increased cell apoptosis rate was detected using the TUNEL and IHC staining assay of Cleaved-caspase 3 in the ovaries of succinate-administered mice in contrast to the control mice (Fig. 5D and E). We observed positive TUNEL and Cleaved-caspase 3 staining at ovarian cells and mainly in the GCs, indicating that the increased apoptosis of GCs is associated with ovarian dysfunction. Importantly, the mRNA and protein levels of proliferation- and apoptosis-related genes in the ovary administered with succinate in vivo were detected by qRT-PCR and western blot. As shown in Fig. 5F, the mRNA expression levels of ovarian cell proliferation and apoptosis genes were significantly affected by high Ksuc stimulation, particularly for *BCL-2* and *CASPASE3*. Meanwhile, obvious changes in Bcl-2 and Cleaved-caspase 3 protein levels were detected in the succinate group compared with the control group (Fig. 5G and H). The changes of these factors were generally consistent with the increased cell apoptosis of ovary cultured in vitro induced by high level of Ksuc, further confirming the role of Ksuc in cell apoptosis of mouse ovary. Moreover, the expression of P21, the aging marker, increased significantly in mouse ovary after succinate administration in vivo (Fig. 5F-H).

## Discussion

Although there were some studies related to PTMs in ovarian aging [4–6], the specific role of PTMs in the pathophysiological mechanisms of ovarian aging is not well clear. Lysine succinylation (Ksuc) is a conserved, novel PTM commonly found in eukaryotes and prokaryotes, which can affect protease activity and gene expression [19]. In this study, we speculated firstly on the possible involvement of Ksuc in the ovarian aging process by increased Ksuc in ovaries of aged and POI mice, and distribution of Ksuc in various types of mice ovarian cells and the high level of Ksuc in GCs.

Ovarian aging is a major factor in female infertility and characterized by decreased follicular quantity and quality together with low levels of AMH and E2 [2, 20]. Therefore, based on preliminary correlation between Ksuc and ovarian aging demonstrated by the above localization and quantitative assays, we further explored the effect of elevated Ksuc level with addition of succinate on ovarian aging determined by follicular atresia, AMH and E2 levels and aging marker expression in the mouse ovary. AMH and E2 are regarded as important indicators of ovarian reserve, which can regulate ovarian follicle development [21, 22]. Additionally, AMH, one factor of the transforming growth factor beta (TGF-β) superfamily, inhibits primordial follicle recruitment and follicular development [23, 24]. Besides, AMH has highest expression in GCs of the preantral and small antral follicles, while there is little AMH expression in atretic follicles [25, 26]. Interestingly, we found that the serum levels of AMH and E2 in the succinate group were significantly decreased along with increased atretic follicles and reduced secondary, antral and total follicles. These results suggest that high Ksuc level likely inhibits the ovarian reproductive and endocrine functions.

P53 can promote cell cycle arrest, apoptosis and senescence by activating its target genes like P21, a cell cycle regulatory protein, which can mediate cellular senescence via P53-dependent and -independent pathways [27–30]. Our study shows that a high level of Ksuc could greatly elevate the expression of the aging marker. However, P53 had no significant difference between the succinate group and the control group (Fig. S4D). In this regard, we here hypothesized that P21 may affect ovarian aging through a P53-independent pathway in our study [31]. In addition, recent research on the interaction between P53 and P21 demonstrated that P21 can promote P53 degradation, which may also account for this result. Further study is acquired to reveal the relationship between P21 and P53 in mice ovarian dysfunction induced by excessive Ksuc.

GCs are essential for follicle development and homeostasis because they provide nutrients and mechanical support for oocytes. And excessive apoptosis of GCs can damage the oocyte quality and decrease hormone production [22, 32]. In addition, GCs also regulate the activity of oocyte at transcriptional level and facilitate the PTM of many oocyte proteins [33]. Therefore, the PTMs in GCs associated with its apoptosis ability may be the important way for exploring the mechanism of ovarian aging. We found that ovarian cells especially GCs apoptosis significantly in succinate groups not only in vitro but also in vivo by TUNEL and IHC assays of Cleaved-caspase 3, the apoptotic execution protein [34]. As revealed in our IHC analysis of Ksuc in mice ovary, there are high levels of Ksuc in GCs with increased atretic follicles in mice ovaries of succinate groups in vitro and in vivo, which is likely the result of high Ksuc level promoting follicular atresia through affecting the GCs dysfunction. Previous research has revealed that there are total of 2565 succinylation sites corresponding to 779 proteins in mammal cells and succinylated lysines are highly enriched in both Mt and extra-Mt proteins which regulate cell proliferation, oxidative damage and apoptosis [8, 10, 35]. Taken together, we predict that Ksuc may regulate the mitochondrial function, proliferation and apoptotic function of GCs, which requires further exploration of the succinylation modification sites to clarify the linkage. In addition, the expression of proliferation and differentiation markers in GCs during folliculogenesis, including follicle stimulating hormone receptor (FSHR), Lutropin-choriogonadotropic hormone receptor (LHCGR), cytochrome P450 11A1 (CYP11A1) and cytochrome P450 19A1 (CYP19A1) of mouse ovary administered in vivo [36, 37], were examined. *AMH*, *LHCGR* and *CYP11A1* mRNA levels were significantly decreased in the succinate group (Fig. S4D). These data indicate that preovulatory GCs did not properly proliferate and differentiate in response to the elevated Ksuc level.

In addition, bone morphogenetic protein 15 (BMP-15) and growth differentiation factor 9 (GDF-9), members of the TGF-β family, also play a key role in follicular development by influencing the proliferation and differentiation of GCs and their communication with the oocyte [38, 39]. These growth factors are responsible for regulation of mRNA expression of follicle-stimulating hormone (FSH) [40] and luteinizing hormone (LH) [41] receptors in growing follicles. We also observed that the mRNA expression of LH receptor was positively correlated with the mRNA expression of *BMP15* in mouse ovary (Fig. S4D), which suggests that there are interactions between Ksuc, the genes mentioned above and follicular development during ovarian aging.

## Conclusion

**Fig. 6:**
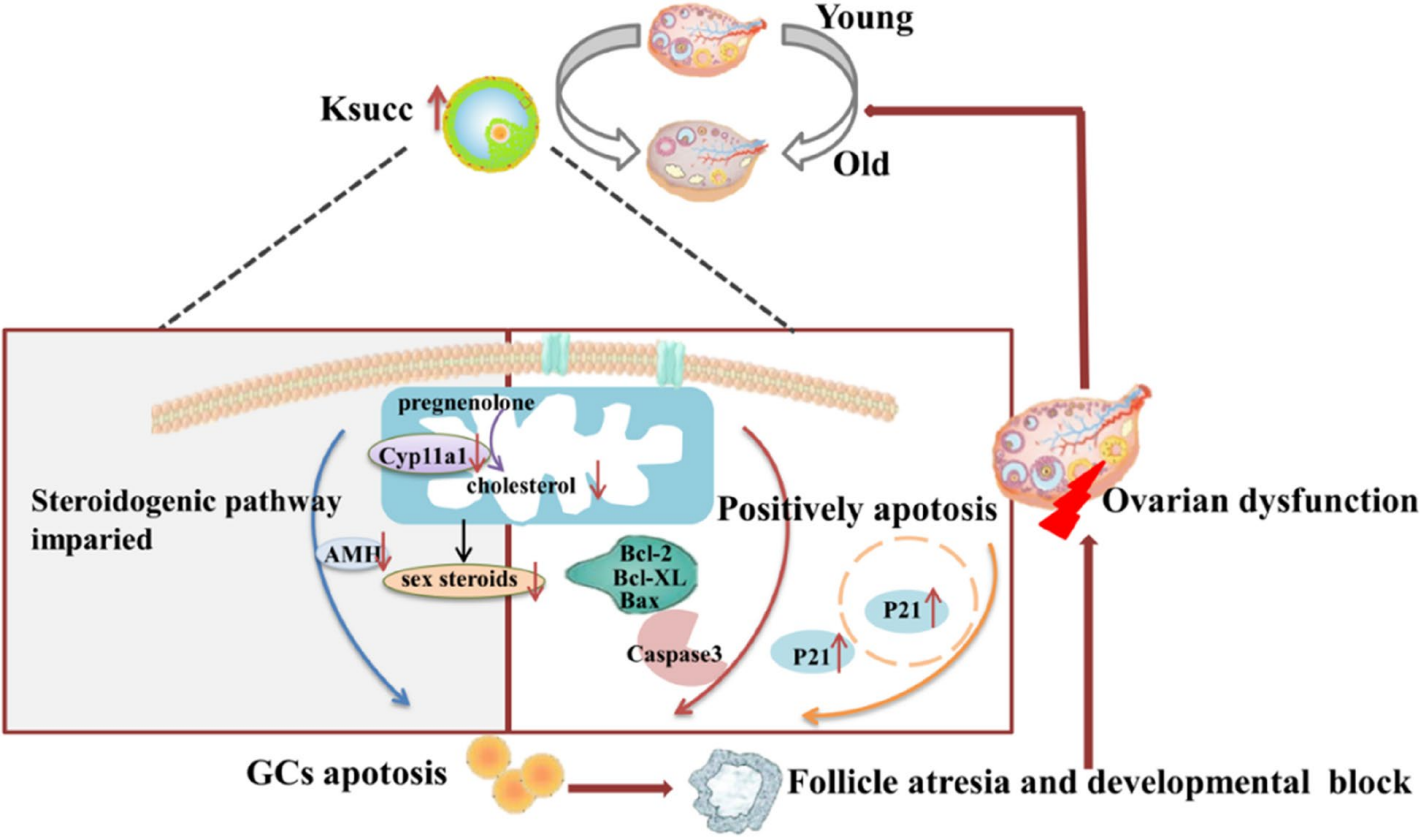
The simple regulatory model of Ksuc in ovarian aging

The present study has identified a newly-discovered PTM (Ksuc) in mouse ovary and found that high Ksuc level most likely contributes to ovarian aging in combination with increases in markers of cellular aging, ovarian cell apoptosis, and suppression of proliferation and steroidogenic events, which may provide potential biomarkers for ovarian aging (Fig. 6).

## Supplementary Information

**Additional file 1:** **Figure S1. **Validation of successful mouse POI model construction. (A) and (B) Histopathological examination and follicle counts of ovaries. Scale bar: 200 μm. (C) and (D) Ovary weight (mg) and ovary index (ovary weight / body weight, mg/g). (E) and (F) Levels of serum AMH and E2 concentrations. * *P* < 0.05, ** *P* < 0.01.

**Additional file 2:** **Figure S2.** (A) The negative control of IHC-Ksuc and -Cleaved Caspase 3 in mouse ovary administered *in vivo* sections. Scale bar: 100 μm (B) The negative control of IF-Ksuc (green) and -DAPI (blue) in mouse ovarian sections. Scale bar: 100 μm.

**Additional file 3:** **Figure S3.** The negative control and positive control of TUNEL staining in mouse ovarian sections. Scale bar: 200 μm.

**Additional file 4: Figure S4.** (A) The occurrence and distribution of Ksuc in mouse ovary were examined by immunofluorescence (IF) assays. Scale bar: 100 μm (B) The level of global lysine acetylation (Kac) of ovaries in succinate and control groups. (C) Western blot analysis of ovarian global lysine malonylation (Kmal) level of ovary in succinate compared with control group. (D) Relative quantitative transcription of ovarian function-related and folliculogenesis genes and P53, an ovarian aging-related marker of mice ovaries administrated with Nacl and succinate in vivo. Error bars: SEM; (*n* = 5 mice / group). * *P* < 0.05, ** *P* < 0.01, *** *P* < 0.001.

**Additional file 5.**

**Additional file 6.**

**Additional file 7.**

## Data Availability

Not applicable.

## Abbreviations

Ksuc: Lysine succinylation
PTM: Post-translational modification
Kac: Lysine acetylation
Kme: Lysine methylation
POI: Premature ovarian insufficiency
NOA: Normal ovarian aging
AMH: Anti-Müllerian hormone
E2: Estradiol
ELISA: Enzyme-linked immunosorbent assay
Mt: Mitochondrial
TCA: Tricarboxylic acid
GCs: Granulosa cells
CTX: Cytoxan
BU: Busulfan
HE: Hematoxylin–eosin
IHC: Immunohistochemistry
IF: Immunofluorescence
BSA: Bovine serum albumin
TUNEL: Transferase-mediated dUTP-biotin nick end labeling
RIPA: Radio-immunoprecipitation assay
PMSF: Phenylmethylsulfonyl fluoride
PVDF: Polyvinylidene fluoride
HRP: Horseradish peroxidase
qRT-PCR: Quantitative real-time PCR
Kmal: Lysine malonylation
Bcl-2: B-cell lymphoma-2
Bax: BCL2-associated X
PCNA: Proliferating cell nuclear antigen
FSHR: Follicle stimulating hormone receptor
LHCGR: Lutropin-choriogonadotropic hormone receptor
CYP11A1: Cytochrome P450 11A1
CYP19A1: Cytochrome P450 19A1
GDF-9: Growth differentiation factor 9
BMP-15: Bone morphogenetic protein 15
TGF-β: Transforming growth factor beta
FSH: Follicle-stimulating hormone
LH: Luteinizing hormone
