# Supplementary figures and images for: The emerging role of lysine succinylation in ovarian aging
Source: Reprod Biol Endocrinol. 2023 Apr 20;21:38. doi: 10.1186/s12958-023-01088-4 (PMC10116721; doi:10.1186/s12958-023-01088-4)

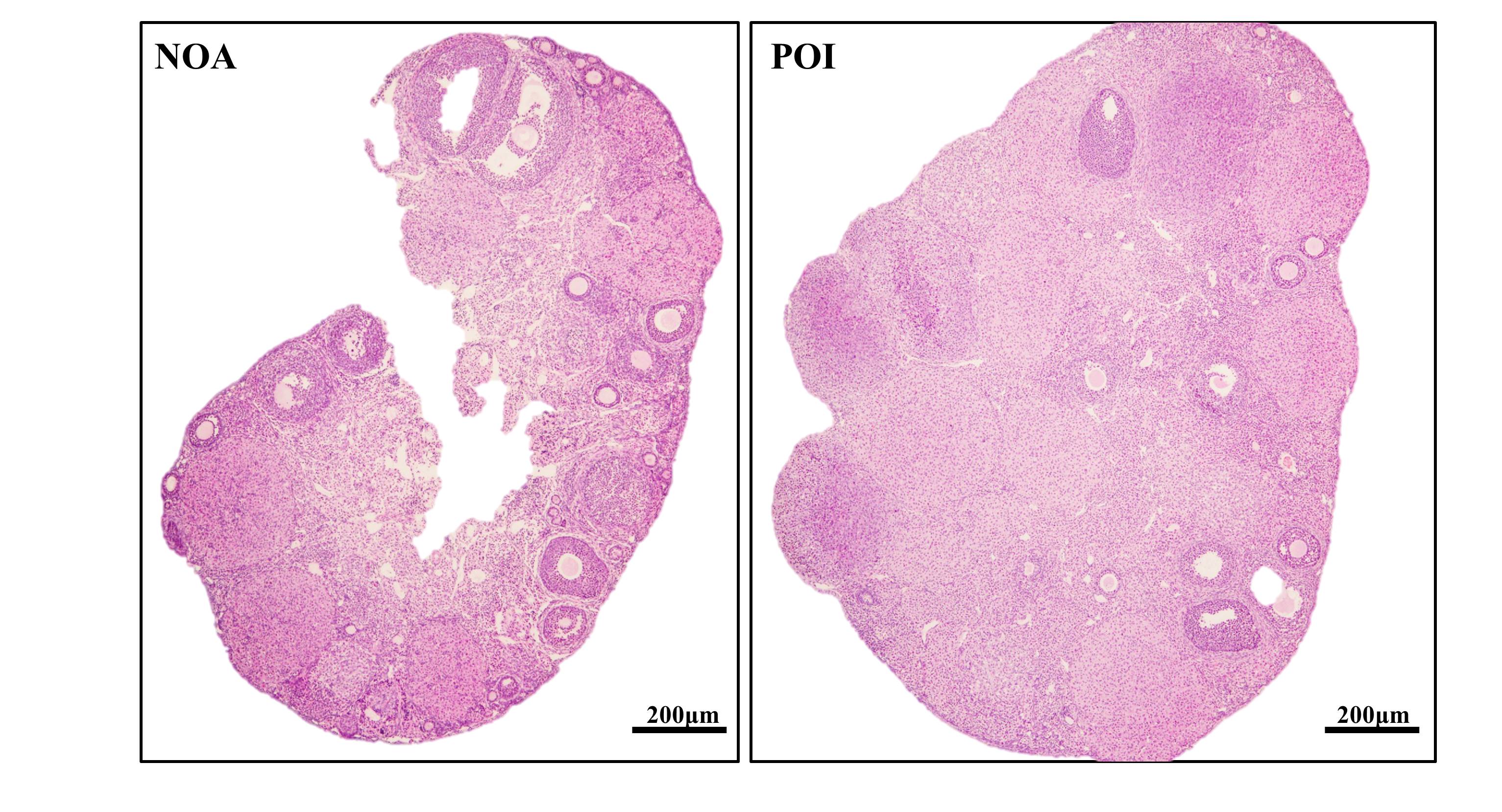

Supplement: Supplementary file 1 — Additional file 1: Figure S1. Validation of successful mouse POI model construction. (A) and (B) Histopathological examination and follicle counts of ovaries. Scale bar: 200 μm. (C) and (D) Ovary weight (mg) and ovary index (ovary weight / body weight, mg/g). (E) and (F) Levels of serum AMH and E2 concentrations. * P < 0.05, ** P < 0.01. [file 12958_2023_1088_MOESM1_ESM.jpg]

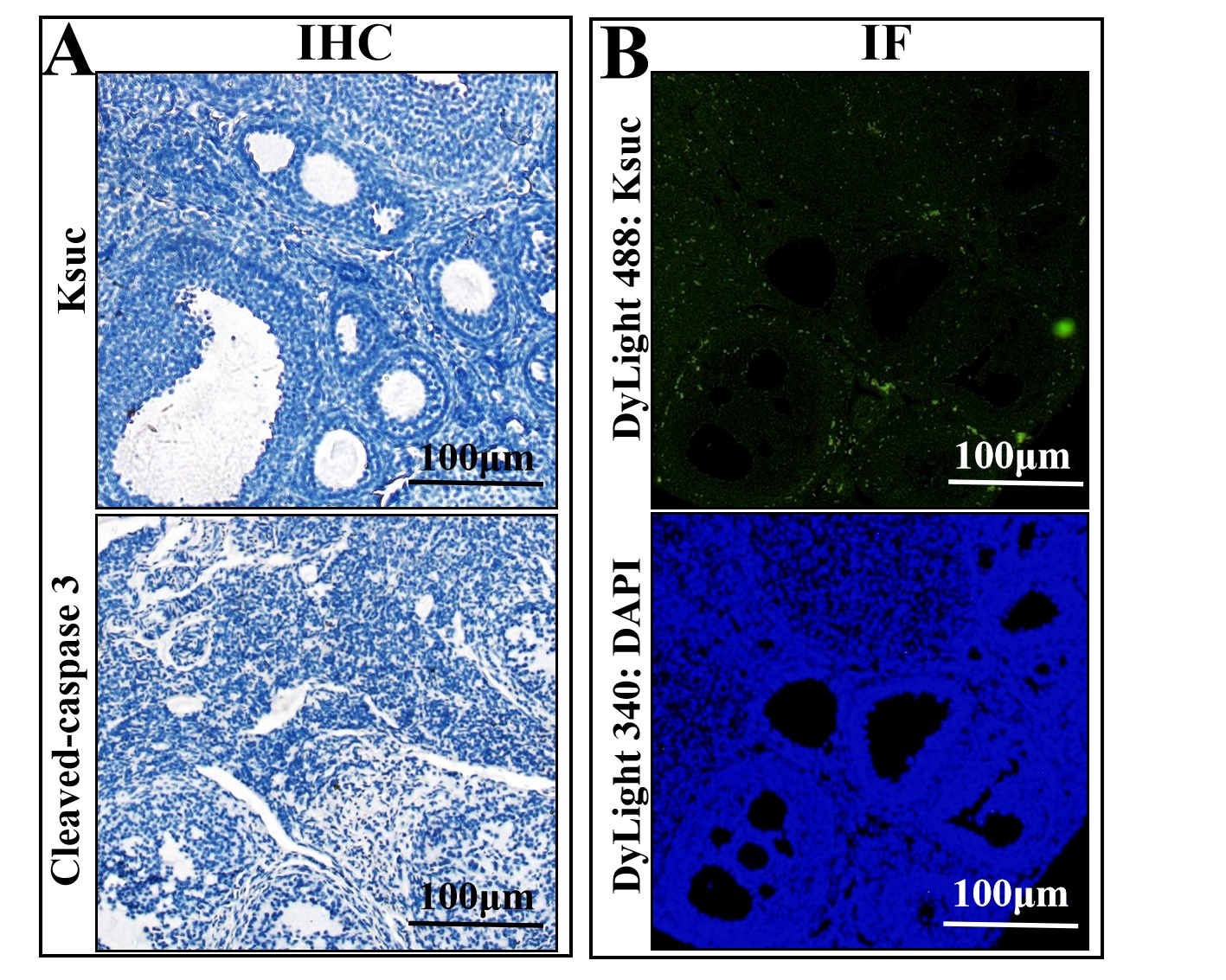

Supplement: Supplementary file 2 — Additional file 2: Figure S2. (A) The negative control of IHC-Ksuc and -Cleaved Caspase 3 in mouse ovary administered in vivo sections. Scale bar: 100 μm (B) The negative control of IF-Ksuc (green) and -DAPI (blue) in mouse ovarian sections. Scale bar: 100 μm. [file 12958_2023_1088_MOESM2_ESM.jpg]

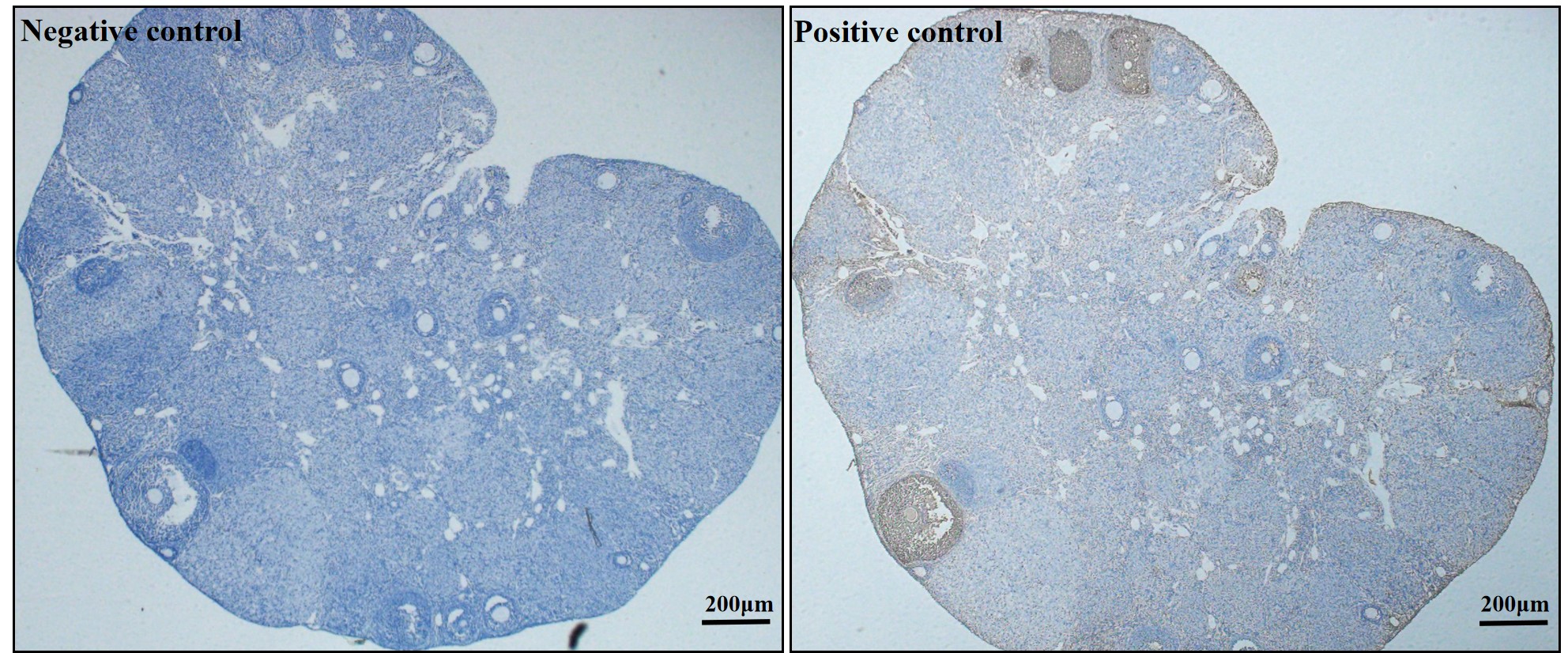

Supplement: Supplementary file 3 — Additional file 3: Figure S3. The negative control and positive control of TUNEL staining in mouse ovarian sections. Scale bar: 200 μm. [file 12958_2023_1088_MOESM3_ESM.jpg]

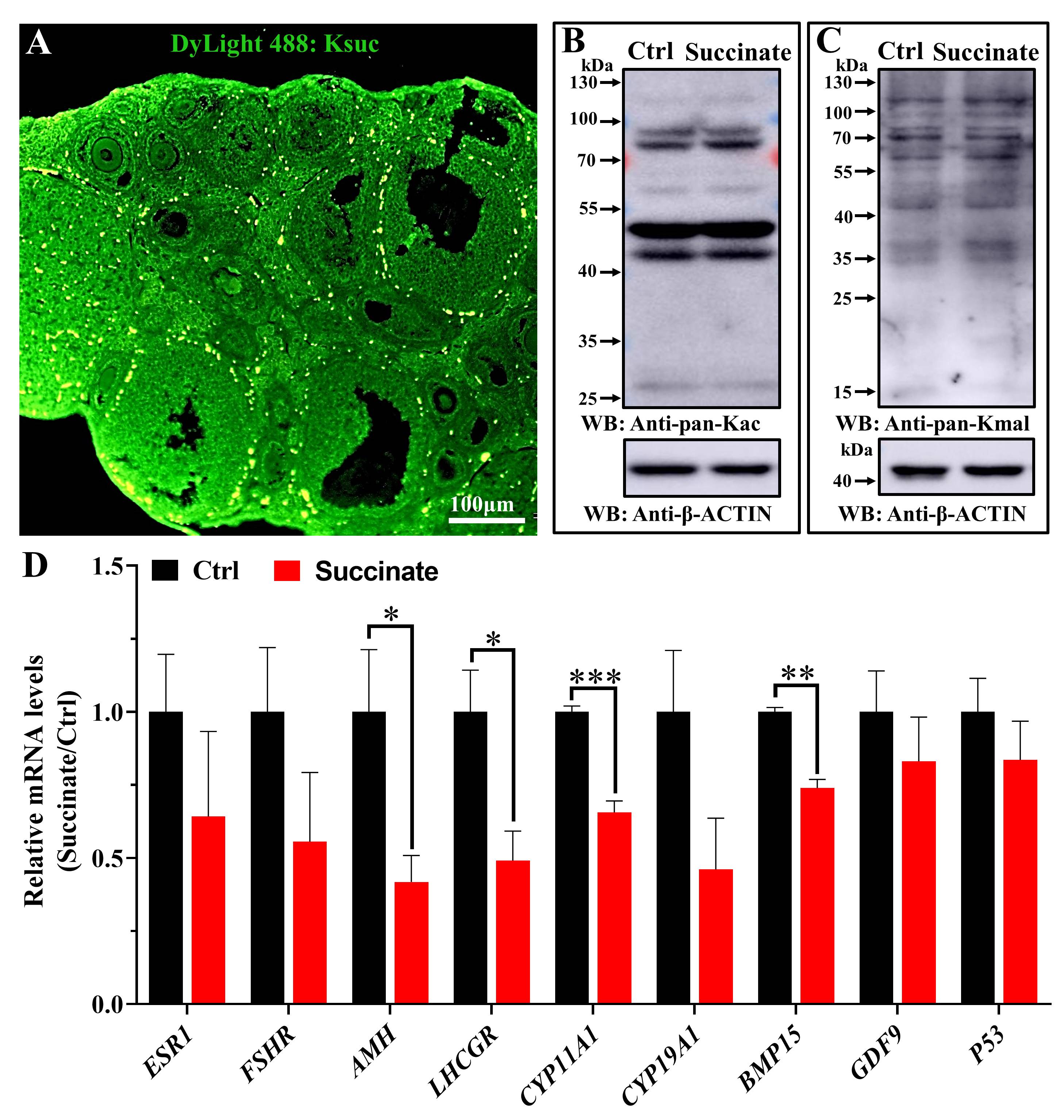

Supplement: Supplementary file 4 — Additional file 4: Figure S4. (A) The occurrence and distribution of Ksuc in mouse ovary were examined by immunofluorescence (IF) assays. Scale bar: 100 μm (B) The level of global lysine acetylation (Kac) of ovaries in succinate and control groups. (C) Western blot analysis of ovarian global lysine malonylation (Kmal) level of ovary in succinate compared with control group. (D) Relative quantitative transcription of ovarian function-related and folliculogenesis genes and P53, an ovarian aging-related marker of mice ovaries administrated with Nacl and succinate in vivo. Error bars: SEM; (n = 5 mice / group). * P < 0.05, ** P < 0.01, *** P < 0.001. [file 12958_2023_1088_MOESM4_ESM.jpg]
