# Supplementary material for: The emerging role of lysine succinylation in ovarian aging
Source: Reprod Biol Endocrinol. 2023 Apr 20;21:38. doi: 10.1186/s12958-023-01088-4 (PMC10116721; doi:10.1186/s12958-023-01088-4)
Supplement: Supplementary file 5 — Additional file 5. [file 12958_2023_1088_MOESM5_ESM.docx]

**Table S1**

| **Animal Use Permit** | |
| --- | --- |
| **License No.:** | **SYXK (gan)-2021-0004** |
| **Name of the entity:** | **Nanchang University** |
| **Legal representative:** | **Changbing Zhou** |
| **Facility Address:** | **Medical Laboratory Animal Center, Nanchang University, No. 71 East Yangming Road, Nanchang city** |
| **Application:** | **barrier environment** |
