# Supplementary material for: The emerging role of lysine succinylation in ovarian aging
Source: Reprod Biol Endocrinol. 2023 Apr 20;21:38. doi: 10.1186/s12958-023-01088-4 (PMC10116721; doi:10.1186/s12958-023-01088-4)
Supplement: Supplementary file 6 — Additional file 6. [file 12958_2023_1088_MOESM6_ESM.docx]

**Table S2**

| **The information of primary antibodies used in western blot** | | |
| --- | --- | --- |
| **Antibodies** | **dilution ratio** | **Origin** |
| **Ksuc** | **1:1000** | **PTMbio (PTM419)** |
| **Kmal** | **1:1000** | **PTMbio (PTM901)** |
| **Kac** | **1:1000** | **PTMbio (PTM-105RM)** |
| **Bcl2** | **1:2000** | **Abcam (ab182858)** |
| **Bax** | **1:5000** | **Proteintech (50599-2-Ig)** |
| **Cleaved–Caspase 3** | **1:2000** | **Cell Signaling Technology (#9664)** |
| **PCNA** | **1:2000** | **Proteintech (10205-2-AP)** |
| **P21** | **1:1000** | **Abclonal (A21061)** |
| **β-Actin** | **1:3000** | **Proteintech (20536-1-AP)** |
