# Supplementary material for: The emerging role of lysine succinylation in ovarian aging
Source: Reprod Biol Endocrinol. 2023 Apr 20;21:38. doi: 10.1186/s12958-023-01088-4 (PMC10116721; doi:10.1186/s12958-023-01088-4)
Supplement: Supplementary file 7 — Additional file 7. [file 12958_2023_1088_MOESM7_ESM.docx]

**Table S3**

| **Gene** | **Primer (5′–3′)** | |
| --- | --- | --- |
|  | **Forward** | **Reverse** |
| ***β-ACTIN*** | **GCGGACTGTTACTGAGCTGC** | **CGCCTTCACCGTTCCAGTTT** |
| ***BCL2*** | **GCTACCGTCGTGACTTCGC** | **CCCCACCGAACTCAAAGAAGG** |
| ***BAX*** | **CCTCCTTTCCTACTTCGG** | **GGTTTATTGGCACCTCCC** |
| ***CASPASE3*** | **GGGGAGCTTGGAACGCTAA** | **CCGTACCAGAGCGAGATGAC** |
| ***PCNA*** | **TTGCACGTATATGCCGAGACC** | **GGTGAACAGGCTCATTCATCTCT** |
| ***CYP19A1*** | **CGCAGAGTATCCAGAGGTCG** | **CGCATGACCAAGTCCACAAC** |
| ***CYP11A1*** | **CCAACCTTTCCTGAGCCCTAC** | **AAACTGACTCCAAAGTGCCCA** |
| ***BMP15*** | **TGGGTTCCTAAATGCCGGAC** | **TGCCTTTTTGCTGCTGACAC** |
| ***GDF9*** | **AGTTCTCCCAAGTCATGGCAC** | **GGCTCCACTCTGGGATTCTTC** |
| ***ESR1*** | **ACCGAAAACACAAGGCTCCC** | **AGAGAGGAACACGGGATGTG** |
| ***AMH*** | **TGGCTGAAGTGATATGGGAGC** | **TAGCACCAAATAGCGGGTGTC** |
| ***FSHR*** | **GAATCCCTGTTCCTCGGCTC** | **CCCTGACCTATCTGCCATGC** |
| ***LHCGR*** | **ACGAGACGCTTCATCACTCTG** | **GATGGCATGTCTCAGCCTCA** |
| ***P21*** | **CCCGAGAACGGTGGAACTTT** | **GAGTGCAAGACAGCGACAAG** |
| ***P53*** | **TCCGAAGACTGGATGACTGC** | **GATCGTCCATGCAGTGAGGT** |
